# Arsenic and Selenium Profile in Erythrocytes of Renal Transplant Recipients

**DOI:** 10.1007/s12011-019-02021-w

**Published:** 2019-12-23

**Authors:** Aleksandra Wilk, Barbara Wiszniewska

**Affiliations:** Department of Histology and Embryology, Pomeranian Medical University in Szczecin, Powst. Wlkp. 72, 70-111 Szczecin, Poland

**Keywords:** Mycophenolate mofetil, Arsenic, Selenium, Renal recipients, Immunosuppressive drug

## Abstract

Arsenic and selenium elements play extremely important roles in organisms. Too high As concentration in blood may lead to functional disorders within organs, including cancer. Arsenic is designated as a Group 1 human carcinogen by the International Agency for Research on Cancer that has established causal role of arsenic in cancers of the urinary bladder, lung, and skin in humans. In contrast, Se is believed to be the antioxidant trace element that is important in the biological defense against oxidative damage. We tested the hypothesis that immunosuppressive treatment based on mycophenolate mofetil (MMF), that is one of the most commonly used drug by renal transplant recipients, affects arsenic and selenium concentration in erythrocytes of renal transplant recipients. Current research was undertaken due to the fact that there are few studies on the concentration of chemical elements in the erythrocytes in kidney patients receiving immunosuppressive drugs. Monitoring of the concentration of chemical elements in the blood in patients who underwent kidney transplantation could be helpful, since chemical elements play an important role in many biological processes and it seems to be crucial in the prevention of cancer to which renal transplant recipients are more often exposed.

The material consisted of blood from 115 renal transplant recipients of the Department of Nephrology, Transplantology, and Internal Medicine of Independent Public Clinical Hospital No. 2, Pomeranian Medical University, in the city of Szczecin in northwestern Poland. Arsenic and selenium levels in erythrocytes were quantified by inductively coupled mass spectroscopy.

Men MMF+ had significantly higher As concentration than men MMF−. Se concentration was significantly higher in younger patients compared with older patients. The patients with lower creatinine level who used MMF had significantly higher As than MMF− patients. Patients whose therapy was based on MMF, cyclosporine A and glucocorticosteroids exhibited significantly higher concentration of As compared with patients whose regimen was based on MMF, tacrolimus, and glucocorticosteroids.

This is the first study that demonstrates that regimen based on mycophenolate mofetil affects As and Se concentrations in erythrocytes in renal transplant recipients.

## Introduction

Renal transplant recipients exhibit decreased immunity due to the intake of immunosuppressive treatment. The use of immunosuppressive drugs is necessary in their case, since it prevents the graft rejection. One of the most commonly used immunosuppresive drugs is mycophenolate mofetil (MMF) which is the mycophenolic acid (MPA) derivative [1]. This is an immunomodulatory drug that inhibits inosine monophosphate dehydrogenase (IMPDH). The drug inhibits the proliferation of T and B cells [2–5]. MMF is mostly codrug with calcineurin inhibitors to prolong allograft function; it is believed that MMF exhibits antioxidant properties [5]. However, on the other side, data on its mechanism of action remain controversial and the following subject definitely need more thorough and in-depth research.

The mechanism of action of MMF needs more studies; however, it is known that it can influence chemical element concentration. Arsenic (As) is involved in oxidative stress that may lead to renal graft disorder. Numerous studies have documented blood As level due to the influence of diet and exogenous factors on its concentration. Sediment and drinking water contaminations with As are global problems. In fish and seafood, arsenic is changed to arsenobetaine and numerous studies concerning the As level in the aspect of fish or/and seafood consumption [6–10]. However, little is known about its concentration in erythrocytes of renal recipients taking into account the influence of immunosuppressive drug intake, including MMF. It seems to be reasonable to monitor arsenic concentration in patients who have undergone renal transplantation due to the fact that As may lead to cancer development [11]. Arsenic is designated as a Group 1 human carcinogen by the International Agency for Research on Cancer (IARC), and IARC has established causal role of arsenic in cancers of the urinary bladder, lung, and skin in humans [12]. It should be emphasized here that organ recipients belong to high-risk cancer patients [13, 14].

In contrast, selenium (Se) is a micronutrient of supreme importance for human health. Numerous studies have shown that the deficiency of this element is to be associated with, among others, diseases resulting from reduced immunity [15, 16]. Se is believed to be the antioxidant trace element that is important in the biological defense against oxidative damage [17, 18]. Selenium fulfills several relevant functions by being comprised (as selenocysteine) of the active sites of proteins called selenoproteins [10]. Additionally, Se is a trace element that plays a significant role in the functions of the immune system and is involved, next to kidneys, in detoxification process [15]. The kidney is responsible for maintaining the homeostasis of numerous trace elements [19, 20]. It has been documented that patients with chronic kidney disease exhibit lower plasma selenium concentration compared with healthy ones [19, 21].

It has been shown that an immunosuppressive regimen based on MMF affects serum trace elements concentration [22, 23]. MMF increases the concentration of Cu and Zn in the blood serum and also reduces the concentration of Na [22]. In addition, MMF disturbs Fe levels in blood [22]. Furthermore, immunosuppressive drugs intake may lead to altered heavy metal level in renal tissue, which has been already documented in our previous studies [24]. Moreover, we observed and documented decreased levels of vanadium and copper in renal grafts [25, 26]. Kidney transplant recipients, besides drugs, are exposed to many various external factors that may lead to various organ dysfunction. It is important to understand the risk factors for chronic kidney disease that lead to renal transplantation, and moreover, to broaden knowledge on the effects of immunosuppressive drugs on the level of chemical elements that seem to be crucial and significant for proper functioning of the organs/tissues.

Arsenic and selenium elements play extremely important roles in organisms; thus, among others, both of them are associated with oxidative stress. However, data regarding the association between MMF intake and As and Se blood level are scarce. Current research was undertaken due to the fact that there are few studies on the concentration of chemical elements in the erythrocytes in kidney patients receiving immunosuppressive drugs. Monitoring of the concentration of chemical elements in the blood in patients who underwent kidney transplantation could be helpful, since, as mentioned above, (i) chemical elements play an important role in many biological processes and (ii) it seems to be crucial in the prevention of cancer [27–29] to which renal transplant recipients are more often exposed.

In order to better understand how the As and Se concentration in erythrocytes of renal recipients changes, we hypothesized that MMF, the most commonly ID used by RTRs, influences and changes the proportions between the aforementioned chemical elements. Therefore, the aim of this current study was to examine and compare the concentrations of arsenic and selenium in erythrocytes of renal transplant recipients treated with MMF and without MMF, in various drug combinations. The second clinical aim was to examine if gender, age, creatinine level, and codrug intake affect the level of As and Se in erythrocytes of RTRs. Furthermore, the correlation between As and Se was determined; thus, it may be related to the immune system defense in aspect of oxidative stress.

## Materials and Methods

### Study Population

The current work described has been carried out in accordance with The Code of Ethics of the World Medical Association (Declaration of Helsinki) for experiments involving humans. The study was approved by the Bioethics Committee of the Pomeranian Medical University (decision KB-0012/74/17).

The material consisted of blood samples from 115 renal transplant recipients (54 women and 61 man, aged 51.2 ± 13.1) of the Department of Nephrology, Transplantology, and Internal Medicine of Independent Public Clinical Hospital No. 2, Pomeranian Medical University, in the city of Szczecin in northwestern Poland, whose function of the graft was stable for over 6 months (creatinine level 1.61 ± 0.88). All patients included in the current studies obtained written information describing the research, including the aims of the study and they agreed to participate in the study. A 10 cc blood sample was obtained during the diagnostic workup and was collected into tubes certified for quantification of As and Se (Vacutainer System, royal blue cap). Blood samples were centrifuged within 30 and 120 min of collection to separate serum from cellular fraction. The samples with erythrocytes were stored at − 80 °C until the arsenic and selenium assays were performed. The patients were divided into two groups: (i) MMF+, patients using mycophenolate mofetil in their regimen and (ii) a control group (MMF−) of patients without MMF in drug protocol. Additionally, our analysis considered gender, age, creatinine level, and codrug intake. Serum creatinine level was obtained as a routine control examination.

### Elements Assay

Arsenic and selenium levels in erythrocytes were quantified by inductively coupled mass spectroscopy (ICP-MS NexION 350D, Perkin Elmer) using methane to reduce polyatomic interferences, previously described by Lubinski et al. [16]. Each sample was measured in duplicates in different analytical runs. Prior to analysis, all samples were centrifuged (6000 rpm, 15 min) and the supernatant was diluted 100 times with the reagent blank.

### Statistical Analysis

The statistical analysis employed StatSoft Statistica 13.3 software and Microsoft Excel 2015. Arithmetic mean (AM), standard deviation of AM (SD), median (Med), and percent coefficient of variation (CV) were established for the concentrations of As and Se. To evaluate the compliance of the results with the expected normal distribution, Kolmogorov–Smirnov (KS) tests with the Lilliefors correction were used (*p* < 0.05). In addition, the mean concentrations of As and Se in erythrocytes were compared between different patients groups. As the data distribution was not consistent with the expected normal distribution, the Mann–Whitney *U* test was used (MWU; *p* < 0.05). Spearman’s rank correlation coefficients (*r*_s_) were also determined.

## Results

Both the K-S test and the K-S test with Lilliefors correction showed no characteristics of normal distribution, and therefore the mean concentrations of metals in the samples were compared with the use of a non-parametric M-W *U* test. To compare the obtained results, medians were used. The number of patients within the groups were different, depending on the aspect of comparison.

### Arsenic and Selenium Concentration in Erythrocytes in All Patients

Taking into account the As and Se levels in all examined patients in the aspect of MMF-based regimen, the concentration levels of both elements were significantly different. Patients who used MMF had significantly higher As level and lower level of Se compared with MMF− patients (Table 1).

**Table 1:** Arsenic and selenium concentrations in erythrocytes of renal transplant recipients

As (μg/L)		
MMF+-based regimen	AM ± SD	2.65 ± 3.67
	Median	1.38*
	Min–max	0.53–23.26
	CV	138.68
Regimen excluded MMF	AM ± SD	1.64 ± 1.76
	Median	1.01*
	Min–max	0.57–7.49
	CV	106.74
	U M-W	*p =* 0.03
Se (μg/L)		
MMF+-based regimen	AM ± SD	103.65 ± 21.68
	Median	99.41*
	Min–max	56.36–181.83
	CV	20.92
Regimen excluded MMF	AM ± SD	114.65 ± 24.81
	Median	111.79*
	Min–max	76.31–172.42
	CV	21.64
	U M-W	*p = 0.04*

*A*, arithmetic mean; *SD*, standard deviation; *CV*, coefficient of variation in %; *p < 0.05, statistically significant difference; *U M-W*, Mann Whitney *U* test

### The Impact of MMF Intake on As and Se Levels in Erythrocytes, by Sex

Regarding general division of all patients included in the current study by sex, we found statistically significant difference in Se level between women and men (*p* = 0.0005) and the values were 110.73 μg/L and 96.46 μg/L, respectively (Table 2). The lowest Se concentration was noticed in the men group and the value was 56.36 μg/L. Additionally, men MMF+ had significantly higher As concentration than men MMF− and the values were 1.34 μg/L and 0.81 μg/L, respectively (*p* = 0.008) (Table 2). No significant difference in As and Se levels between women MMF+ versus MMF− was observed.

**Table 2:** Concentrations of As and Se in μg/L, by sex

	Women (*n* = 54)		Men (*n* = 61)	
As (μg/L)				
AM ± SD Median Min-Max CV	2.51 ± 3.51 1.39 0.53–23.26 139.41		2.35 ± 3.27 1.26 0.57–18.13 138.84	
U MW, NS				
	Regimen included MMF (*n* = 40)	Regimen excluded MMF (*n* = 14)	Regimen included MMF (*n* = 50)	Regimen excluded MMF (*n* = 11)
AM ± SD Median Min-max CV	2.65 ± 3.87 1.41 0.53–23.26 145.94	2.11 ± 2.21 1.28 0.59–7.49 104.19	2.64 ± 3.54 1.34* 0.62–18.13 134.05	1.05 ± 0.63 0.81* 0.57–2.84 60.46
	*U MW, NS*		*p* = 0.008	
Se (μg/L)				
AM ± SD Median Min-max CV	112.88 ± 22.63 110.73* 76.02–181.83 20.04		99.98 ± 21.25 96.46* 56.36–178.61 21.25	
*p* = 0.0005				
	Regimen included MMF (*n* = 40)	Regimen excluded MMF (*n* = 14)	Regimen included MMF (*n* = 50)	Regimen excluded MMF (*n* = 11)
AM±SD Median Min-max CV	110.64 ± 21.68 108.47 76.02–181.83 19.59	119.28 ± 24.87 118.37 76.31–157.48 20.85	98.05 ± 20.21 96.33 56.36–178.61 20.60	108.76 ± 24.61 102.08 87.11–172.42 22.62
	U MW, NS		U MW, NS	

*AM*, arithmetic mean; *SD*, standard deviation; *CV*, coefficient of variation in %; *p < 0.05, statistically significant difference; *UM-W*, Mann Whitney *U* test)

### The Impact of MMF Intake on As and Se Levels in Erythrocytes, by Age

In comparing As concentration between younger and older patients, patients under 50 years of age displayed lower As level than patients above 50 years old and the values were 1.22 μg/L vs. 1.38 μg/L. The maximum value was observed in older patients group and it was 23.26 μg As/L. The maximum value was over twice higher than the maximum value in younger patients group. No significant differences in As levels were noticed between the aforementioned groups in aspect of MMF intake (Table 3).

**Table 3:** Concentrations of As and Se in μg/L, by age

	Patients > 50 years old (*n* = 72)		Patients < 50 years old (*n* = 43)	
As (μg/L)				
AM±SD Median Range CV	2.66 ± 3.93 1.38 0.57–23.26 147.62		2.04 ± 2.12 1.22 0.53–10.23 103.81	
U MW, NS				
	MMF-based regimen (*n* = 58)	Regimen without MMF (*n* = 14)	MMF-based regimen (*n* = 32)	Regimen without MMF (*n* = 11)
AM±SD Median Range CV	2.83 ± 4.23 1.51 0.59–23.26 148.99	1.92 ± 2.25 0.92 0.57–7.49 117.31	2.3 ± 2.38 1.29 0.53–10.26 103.29	1.3 ± 0.76 1.02 0.59–2.84 58.63
	U MW, NS		U MW, NS	
Se (μg/L)				
AM±SD Median Range CV	103.41 ± 22.65 98.07* 56.36–181.83 21.91		110.46 ± 22.49 108.29* 66.9–172.42 20.35	
*p =* 0.04				
	MMF-based regimen (*n* = 58)	Regimen without MMF (*n* = 14)	MMF-based regimen (*n* = 32)	Regimen without MMF(*n* = 11)
AM±SD Median Range CV	101.99 ± 23.03 96.81 56.36–181.83 22.58	109.22 ± 20.72 103.59 76.31–149.11 18.97	106.65 ± 18.97 107.01 66.91–165.33 17.79	121.56 ± 28.73 117.25 84.41–172.42 23.64
	U MW, NS		U MW, NS	

*AM*, arithmetic mean; *SD*, standard deviation; *CV*, coefficient of variation in %; *p < 0.05, statistically significant difference; *U M-W*, Mann Whitney *U* test)

Taking into account Se concentration, it was significantly higher in younger patients compared with older patients (108.29 μg/L vs. 98.07 μg/L, respectively, *p* = 0.04) although the highest Se level was confirmed in patient over 50 years old. No significant differences in Se levels were noticed between aforementioned groups in aspect of MMF intake (Table 3).

### The Impact of MMF Intake on As and Se Levels in Erythrocytes, by Creatinine Level

The impact of MMF intake on As and Se levels was also examined in aspect of creatinine level. Our results show that higher concentration of As is present within erythrocytes of patients with creatinine level over 1.6 mg/dL than in patients with lower creatinine level. Additionally, we divided both groups according to the aspect of MMF intake. The patients with lower creatinine level who used MMF had significantly higher As than MMF− patients and these levels were 1.32 μg/L vs. 0.95 μg/L (*p* = 0.03) (Table 4).

**Table 4:** Concentrations of As and Se in μg/L, by creatinine level

	Creatinine level < 1.6 (*n* = 78)		Creatinine level > 1.6 (*n* = 37)	
As (μg/L)				
AM±SD Median Range CV	2.26 ± 3.04 1.22 0.53–23.26 134.49		2.78 ± 3.99 1.55 0.59–18.13 143.38	
U MW, NS				
	Regimen-based on MMF (*n* = 63)	Regimen without MMF (*n* = 15)	Regimen based on MMF (*n* = 27)	Regimen without MMF (*n* = 10)
AM±SD Median Range CV	2.37 ± 3.21 1.32* 0.53–23.26 135.38	1.79 ± 2.21 0.95* 0.57–7.49 123.07	3.28 ± 4.57 1.81 0.62–18.13 139.17	1.43 ± 0.78 1.36 0.59–2.84 54.68
	*p =* 0.03		U MW, NS	
Se (μg/L)				
AM±SD Median Range CV	106.76 ± 22.55 103.79 56.36–181.83 21.12		99.98 ± 21.25 96.46 56.36–178.61 21.25	
U MW, NS				
	Regimen based on MMF (*n* = 63)	Regimen without MMF (*n* = 15)	Regimen based on MMF (*n* = 27)	Regimen without MMF (*n* = 10)
AM±SD Median Range CV	104.45 ± 21.69 101.36 56.36–181.83 20.77	116.45 ± 20.81 117.25 84.41–172.42 20.81	101.78 ± 21.96 96.46 75.86–178.61 21.58	111.95 ± 26.73 105.11 76.31–157.48 23.87
	U MW, NS		U MW, NS	

*AM*, arithmetic mean; *SD*, standard deviation; *CV*, coefficient of variation in %; *p < 0.05, statistically significant difference; *U M-W* Mann Whitney *U* test

The impact of MMF intake on Se levels in erythrocytes, by creatinine level, was additionally determined. Around 10% higher Se level was observed in patients with creatinine level below 1.6 mg/dL than over 1.6 mg/dL (Table 4). No significant differences in Se concentration between aforementioned groups with additional division on MMF+ and MMF− patients (Table 4).

### Codrugs

#### Three-Drug-Based Regimen

##### Mycophenolate Mofetil + Calcineurine Inhibitor + Corticoids

We also examined As and Se levels in the blood of transplant recipients due to codrug intake. Patients whose therapy was based on MMF, cyclosporine A (CsA), and glucocorticosteroids exhibited significantly higher concentration of As compared with patients whose regimen was based on MMF, tacrolimus (Tac), and glucocorticosteroids (G) (2.51 μg/L and 1.3 μg/L, respectively) (Table 5).

**Table 5:** As and Se concentrations in μg/L in MMF+ patients, by intake of a calcineurine inhibitor as a codrug

	MMF + CsA + G (*n* = 6)	MMF + Tac + G (*n* = 48)
As (μg/L)		
AM±SD Median Range CV	4.7 ± 6.21 2.51* 1.43–17.35 129.99	2.64 ± 4.18 1.3* 0.59–23.26 157.87
*p =* 0.04		
Se (μg/L)		
AM±SD Median Range CV	99.65 ± 10.67 100.74 84.91–110.06 10.71	104.49 ± 24.57 100.36 56.36–181.83 23.51
U MW, NS		

*CsA*, cyclosporine A; *Tac*, tacrolimus; *G*, glucocorticosteroid; *U M-W* Mann Whitney *U* test

#### Two-Drug-Based Regimen

##### Mycophenolate Mofetil + Calcineurine Inhibitor

**Fig. 1:**
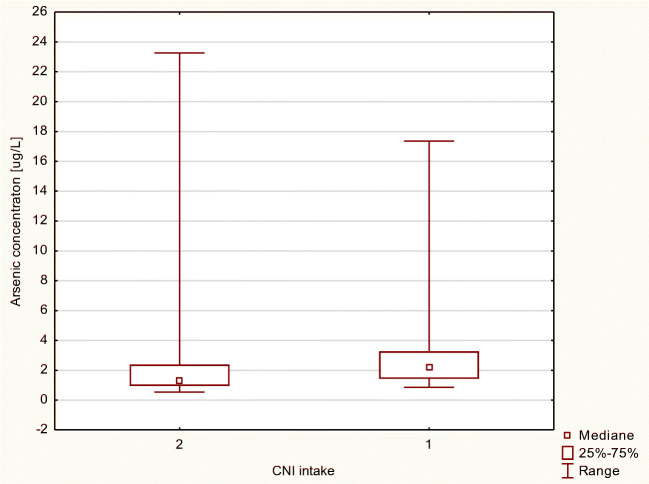
Arsenic concentration in μg/L in MMF+ patients, by intake of a CNI (calcineurine inhibitor) as a codrug (2, tacrolimus; 1, cyclosporine A)

**Fig. 2:**
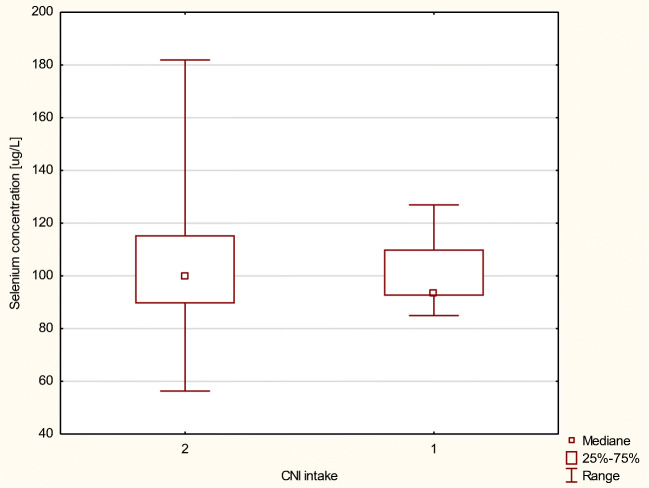
Selenium concentration in μg/L in MMF+ patients, by intake of a CNI (calcineurine inhibitor) as a codrug (2, tacrolimus; 1, cyclosporine A)

We also examined As and Se levels in blood of transplant recipients due to two-drug-based therapy intake. Patients whose therapy was based on MMF and cyclosporine A (CsA) (without corticosteroids) exhibited slightly higher concentration of Se compared with patients whose regimen was based on MMF and tacrolimus (Tac); however, the difference was not statistically significant (Figs. 1 and 2).

##### Mycophenolate Mofetil + Glucocorticosteroids

**Fig. 3:**
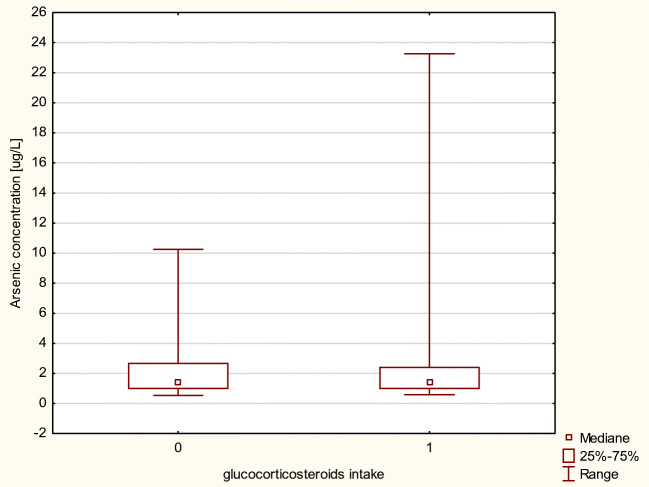
Arsenic concentration in μg/L in MMF+ patients, by intake of a glucocorticosteroids as a codrug (0, regimen without glucocorticosteroids; 1, regimen including glucocorticosteroids)

**Fig. 4:**
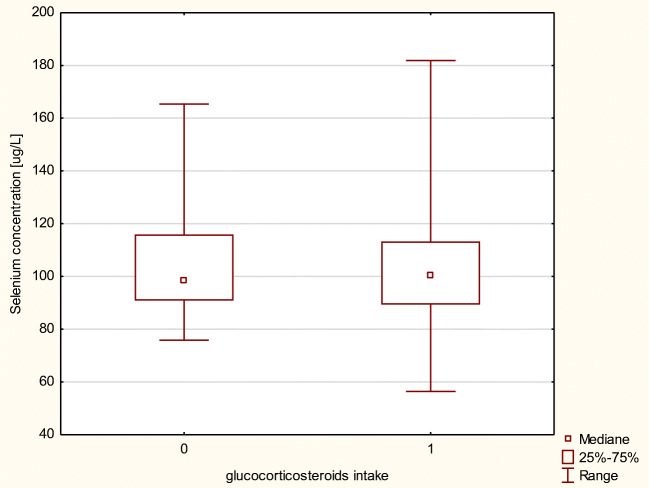
Selenium concentration in μg/L in MMF+ patients, by intake of a glucocorticosteroids as a codrug (0, regimen without glucocorticosteroids; 1, regimen including glucocorticosteroids)

We investigated As and Se levels in the blood of transplant recipients due to glucocorticosteroids as a complementary drug in immunosuppressive therapy. Patients whose therapy was based on MMF and corticosteroids exhibited almost the same level of As and Se compared with patients whose regimen did not include corticoids (Figs. 3 and 4). No statistical differences in element concentrations in erythrocytes between aforementioned groups of patients were observed.

#### mTORs

Regarding mTORs, as codrugs, the number of patients was not numerous enough to obtain reliable statistical analysis (data not shown).

### Correlations Between As and Se Concentrations

Furthermore, Spearman’s rank correlation coefficient indicated there to be a synergistic relationship between As and Se concentrations in all patients (*r*_s_ = 0.25, *p* < 0.05; Table 6). Additional correlation was found in patients treated with MMF (*r*_s_ = 0.31, *p* < 0.05; Table 6).

**Table 6:** Correlation coefficients of As and Se in renal transplant recipients, by MMF intake (*p < 0.05, statistically significant difference)

Correlated elements	Correlation coefficients in		
	MMF+ patients	MMF- patients	All patients
As/Se	0.31*	NS	0.25*

## Discussion

Renal transplant recipients need to use immunosuppressive drugs due to their antirejection properties. Immunosuppressive therapy is mostly based on several drugs that have different mechanisms of action. On the one hand, they prolong the proper function of the transplanted graft; on the other hand, they may cause various side effects and may lead to many disorders, including ion concentration imbalance. Among aforementioned medicines, IMDH inhibitors are distinguished, including mycophenolate mofetil (MMF).

Little is known about arsenic and selenium profiles in erythrocytes of blood of renal transplant recipients. Our results show that both blood As and Se levels significantly differ between patients whose regimen is based on MMF (MMF+) vs. patients who did not use MMF (MMF−). MMF+ patients displayed higher As level and lower Se level vs. MMF− patients. MMF increases As level, which can lead to many pathological alterations within tissues/organs, including transplanted graft. Arsenic toxicity is associated with its reactivity and generation of ROS- and sulfur-containing compounds. The kidney is highly vulnerable to damage caused by ROS, which are involved in the pathogenic mechanism of conditions. Arsenic induces oxidative stress in the kidney and may lead to dysfunction of renal tubular epithelial cells [19]. Furthermore, Zhao et al. [30] investigated the influence of As on inflammation, differentiation of diverse T cell subsets, and the phenotypic molecules and functions of dendritic cells (DCs) [30]. Moreover, chronic arsenic exposure disrupts the immune homeostasis possibly by interfering with the differentiation of Th1/Th2/Th17 subsets as well as the function of DCs [30]. Immunity is a matter of supreme importance in renal transplant recipients even if it needs to be lowered. In contrast, selenium is believed to be an antioxidant nutrient that is important in the biological defense against organs/tissues damage via induction of oxidative stress [19]. It is believed that selenium in the kidney may decrease the levels of the inflammatory cytokines (induced, among others, by heavy metals) [19]. Current data regarding MMF are controversial [2, 5]. As mentioned before, little is known about the effect of MMF on Se and As levels; however, MMF is indeed involved in oxidative stress mechanisms. Ferjani et al. [2] documented that the MMF with tacrolimus-based regimen developed oxidative stress in the spleen of rats, indicated by a significant increase of malondialdehyde and protein oxidation and decrease of antioxidant enzyme levels such as catalase and superoxide dismutase. This damage was associated with an increase of DNA fragmentation [2]. Other results suggest that coadministration MMF with tacrolimus protects the liver and kidney against tacrolimus toxicity via an antioxidant process [5]. We have not confirmed the mechanism of action, since that was not the goal of the current study; however, we revealed that the regimen that include MMF increased As and lowered Se level in renal transplant recipients, which seems to be unfavorable in aspect of function of transplanted organ. Furthermore, Kaminska et al. [22] found that MMF treatment affects plasma Fe, Zn, and Cu levels by increasing plasma Na concentration. All aforementioned results suggest that monitoring of elements, including As and Se may be beneficial.

Our findings were also confirmed regarding the sex and age aspects. Interestingly, men MMF+ had almost twice higher As concentration vs. men MMF−. Considering Se level, significantly higher concentration was observed in women vs. men. Additionally, statistical analysis of our results suggests that the Se level decreases with age. The older group of patients exhibited significantly lower serum Se level than younger ones. The older people exhibit a weaker antioxidant system of defense, since Se, as it was mentioned above, is thought to be antioxidant [31, 32].

The function of renal graft is reflected by, among others, the level of creatinine. Therefore, we divided patients by impact of serum creatinine concentration. In patients with lower creatinine, we observed that MMF significantly increased blood As concentration. Our results seem to be significant, because they reveal, that MMF affects As level in erythrocytes and it correlates with creatinine level. No data were found regarding MMF’s influence on As concentration; however, it is known that the As metabolites and creatinine may share a mechanism of renal secretion. Additionally, total As exposure may be detrimental to renal function. Prospective studies examining the association between As exposure, As methylation capacity, and chronic kidney disease incidence are required [33]. Perhaps MMF increases its level, in < 1.6 creatinine level patients, which seems to be controversial with some other studies recommending MMF as an antioxidative drug used with calcineurine inhibitors.

MMF is commonly used together with calcineurine inhibitor (tacrolimus or cyclosporine); also, the use of this combined drug lowers the incidence of renal dysfunction caused by CNIs. Therefore, we compared blood levels of As and Se in aspect of multidrug therapy including MMF. It turned out that taking into account calcineurine inhibitors and steroids, as codrugs, As level was significantly higher in patients treated with MMF and glucocorticoids with CsA compared with Tac. It seems to be extremely interesting due to the fact that Tac is believed to be much more nephro- and hepatotoxic than CsA and it is used in acute renal graft rejection stage. Ferjani et al. [5] confirmed that the combination of MMF and TAC even at a high dose for the improvement of immunosuppression and the reduction of the side effect of TAC such as nephrotoxicity and hepatotoxicity [5]. It is to be emphasized that perhaps regimen based on MMF and CsA increases As level. Since high concentration of As can only lead to induction of oxidative stress, it cannot be considered an oxidative stress marker.

Arsenic and selenium exhibit, as mentioned above, controversial properties. We found positive correlation between As and Se levels in MMF+ patients and all patients included in the current study. Due to opposite properties of these two elements it, seems to be clear and reasonable.

Among chemical elements, arsenic and selenium seem to be extremely important due to their association with oxidative stress, cancer risk, and/or potential relationship between blood Se and prevalence of stroke [34, 35]. Therefore, our data suggest that monitoring of As and Se may be beneficial. Especially due to the fact that renal transplant recipients belong to the group of high cancer-risk patients.

This is the first study that demonstrates that regimen based on mycophenolate mofetil affects blood As and Se in renal transplant recipients. These data illustrate the need for the monitoring of elements in the blood of the mentioned group of patients and obtained results may be important both for the patients and for the clinicians.
